# Solitary parathyroid adenoma presenting with spine fracture in a paediatric patient

**DOI:** 10.1186/1687-9856-2013-S1-P163

**Published:** 2013-10-03

**Authors:** Marichu P Mabulac, Carol Boongaling, Sioksoan Chan-Cua

**Affiliations:** 1 Section of Pediatric Endocrinology, University of the Philippines- Philippine General Hospital, Manila, Republic of the Philippines

## Background

Solitary parathyroid adenoma is a rare cause of primary hyperparathyroidism in children. We report a case of patient who presented with difficulty in ambulating due to fracture from hypercalcemia.

## Case

A. A. 16 years old, female referred to our service for evaluation of hypercalcemia. The patient presented with fracture at the thoraco-lumbar spine after being accidentally slipped on the floor. She sought consult at a private hospital and an orhtopedic brace was applied. Three months after the incident, the patient complaint of limping and eventually with difficulty in ambulating. She was admitted at the Philippine Orthopedic Center for spinal traction. During her admission, there was an incidental finding of hypercalcemia and the patient was referred to our institution for endocrine evaluation. The patient had history of easy fatigability, palpitations, polyuria, polydipsia, constipation (2x/week), sweating associated with weight loss about half a year prior to her recent admission. On physical examination, patient was ambulatory but limping, not in respiratory distress. Vital signs showed: BP: 100/60, HR: 96/min, RR: 20/min, afebrile. Weight: 28.6 (<2SD), Height: 136.5cm (<3SD), BSA: 1.04, BMI: 15.37, MPH: 145.75cm. Laboratory assessment showed serum calcium 13.3 mg/dL (8.6-10), ionized calcium 2.13 mmol/L (1.10-1.35), serum intact PTH > 2500 pg/mL (15-68.3), phosphorous 1.52 (1.30-2.26), Alkaline Phosphorous 178 IUL (32-91). KUB Ultrasound showed bilateral enlarged kidneys. Thyroid ultrasound revealed a 1.7x1.6cm fluid nodule on the left lobe but the thyroid hormone profile was normal. The Technetium-99m Siestamibi scan showed parathyroid adenoma in the inferior left thyroid bed. The patient underwent total parathyroidectomy with autotransplantation of the remaining parathyroid at (L) forearm. Post-operatively, she had “hungry bone syndrome” due to persistent hypocalcemia 5.5 mg/dL (8.6-10) and urinary calcium/creatinine ratio of less than 0.05 inspite of calcium and calcitriol supplementation. The histopathologic finding was compatible with left parathyroid adenoma of 2.7x2.5x1cm dimension. Five months after the operation, the patient was asymptomatic and the serum phosphorous and alkaline phosphorous are within normal range.

## Conclusion

Delayed diagnosis of hyperparathyroidism in children can often result in significant morbidity. Serum calcium and PTH levels should be checked in children presenting with non-specific symptoms as parathyroid adenoma section is curative and usually restores serum calcium levels back to normal, thereby avoiding further complications.

